# Intermittent fasting triggers interorgan communication to improve the progression of diabetic osteoporosis

**DOI:** 10.1080/19490976.2025.2555619

**Published:** 2025-09-30

**Authors:** Zhiyuan Guan, Wenyu Xiao, Zhiqiang Guan, Jin Xiao, Xuehan Jin, Shengfu Liu, Yin Qin, Liying Luo

**Affiliations:** a Department of Orthopaedics, Shanghai Tenth People’s Hospital Chongming Branch, School of Medicine, Tongji University, Shanghai, China; b Department of Dermatology, Xuzhou Municipal Hospital Affiliated with Xuzhou Medical University, Xuzhou, Jiangsu, China; c Department of Rheumatology and Immunology, Affiliated Municipal Hospital of Xuzhou Medical University, Xuzhou, Jiangsu Province, China; d Department of Orthopedics, Wuxi Ninth People’s Hospital Affiliated to Soochow University, Wuxi, Jiangsu, China; e Department of Ophthalmology, Tongren Hospital, Shanghai Jiao Tong University School of Medicine, Shanghai, China

**Keywords:** Osteoporosis, diabetes mellitus, intermittent fasting, exosome, Christensenellaceae, miR-551b

## Abstract

Diabetic osteoporosis is a disease that seriously affects health, and intermittent fasting is a promising dietary approach to manage diabetes. The objective of our study was to analyze the effects of intermittent fasting on diabetic osteoporosis and its possible mechanisms. Streptozotocin-induced diabetes in mice was treated by intermittent fasting. Micro-CT and Immunostaining techniques were utilized to evaluate glycogen synthesis and morphological changes in the tibia. Gut microbiota analysis involved 16S rRNA gene amplification and sequencing. Liquid chromatography-mass spectrometry was employed, and quantitative real-time PCR assessed gene expression levels. Our study found that intermittent fasting improved blood glucose levels in diabetic mice and simultaneously enhanced cancellous bone microstructure, including BMD, BV/TV, Tb.Th, and Tb.Sp, which was revised by intervention with intermittent fasting. Intermittent fasting increased Christensenellaceae Chr) flora abundance. To further validate the role of Chr in diabetic osteoporosis treated with intermittent fasting, we used a gut microbiota transplanting and elimination experiment and Chr supplementation experiment, and the result found that Chr supplementation improved bone mass and microstructure in diabetic osteoporosis mice. In addition, Christensenellaceae facilitated the release of exosomes, which promote osteoclast activity, and exosome sequencing analysis showed miR551b upregulation in Christensenellaceae-derived exosomes, and the miR551b improves bone parameters in diabetic osteoporosis mice by supplement or inhibiting miR551b experiments. In conclusion, our study highlights the role of intermittent fasting in improving osteoporosis in diabetes by regulating changes in the abundance of Chr in the gut microbiota and improving the exosomes miR551b secreted by Chr, which in turn improves osteoblast activity. These findings provide a mechanism of intermittent fasting in managing osteoporosis via the gut microbiota-bone axis, potentially leading to innovative therapeutic approaches for diabetes-mediated osteoporosis.

## SUMMARY BOX HEADING

* “What is already known on this topic?”

- Diabetes often leads to bone metabolism disorders, and intermittent fasting can have a significant impact on the body’s metabolic state.

* “What this study adds”

- Intermittent fasting led to significant improvements in blood glucose levels and bone mass in diabetic mice, as evidenced by enhanced cancellous bone microstructure parameters such as BMD, Tb.Sp, BV/TV, and Tb.Th.
- Intermittent fasting increased the abundance of Christensenellaceae flora, and KEGG pathway analysis revealed that RNA processing and modifications play a critical role in this process.
- Supplementation with Christensenellaceae-derived exosomes, compared to Lactobacillus-derived exosomes, showed superior effects on bone microstructure. The inhibition of miR-551b and the use of exosome inhibitors like GW4869 further demonstrated the role of miR-551b in bone healing.

* “How this study might affect research, practice or policy”

- Our findings support the mechanism of intermittent fasting in managing osteoporosis via the gut microbiota-exosome-bone axis, potentially leading to innovative therapeutic approaches for diabetes-mediated osteoporosis.

## GRAPHICAL ABSTRACT

**Figure:**
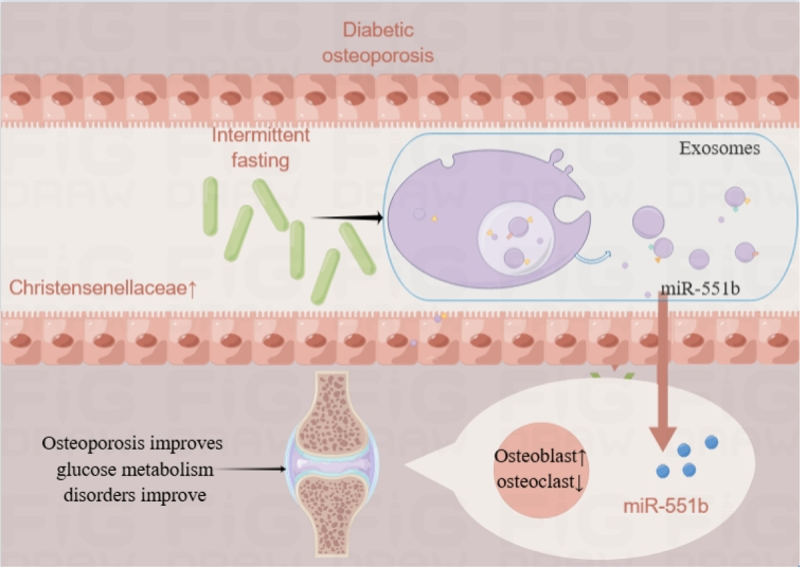

## Introduction

Diabetes mellitus is currently the third leading noncommunicable disease, following cardiovascular diseases and cancer.^1^ Recent studies indicate that 35% of individuals with Type 2 diabetes mellitus experience bone loss, with over 20% diagnosed with osteoporosis.^2,3^ The reinforcing effect that exists between diabetes and osteoporosis significantly accelerates the process of bone loss.^4^ In addition, considering the huge patient base of diabetic patients and osteoporosis patients, diabetic osteoporosis is a difficult problem for human health.^5^ Therefore, understanding the pathophysiology of diabetic osteoporosis and exploring therapeutic options is crucial for improving patient outcomes.

Intermittent fasting(IF), as a lifestyle strategy, has been shown to enhance mitochondrial function and improve insulin sensitivity, along with reducing cardiovascular risk factors in a diet-controlled environment.^6,7^ The metabolic shifts induced by IF increase metabolism and lifespan,^8^ contributing to reduced insulin resistance and obesity.^9–11^ In addition, IF has shown potential in improving both blood glucose control and bone health in diabetic osteoporosis patients. This dietary intervention is particularly relevant given the rising prevalence of diabetes and its associated complications, as diabetes is now the third most dangerous non-communicable disease, trailing only behind cardiovascular disease and cancer.^1^ Numerous bodily systems and organs may be impacted when osteoporosis worsens.^12,13^ The progression of osteoporosis further exacerbates health risks,^4,14^ affecting multiple bodily systems and leading to conditions like fractures and chronic bone pain, which are more prevalent in diabetic bone disease compared to primary osteoporosis.^15,16^

Importantly, IF alters gut microbiota composition, promoting microbial metabolites linked to cognitive function and energy expenditure, while also influencing hippocampal gene expression involved in mitochondrial growth.^17^ These changes significantly impact gut functional pathways and ecological balance, correlating with reduced cardiometabolic risk factors.^18^ The gut microbiota (GM) plays a pivotal regulatory role in energy metabolism and systemic insulin sensitivity, as demonstrated by research in both human and veterinary models of diabetes.^19^ The intricate link between gut microbiota and diet^20^ underscores the importance of microbial balance in maintaining gut health, cognitive function, and brain energy balance.^21^ Furthermore, Exosomes derived from gut microbiota have been found to influence conditions like knee osteoarthritis^22^ through mechanisms involving ferroptosis and the release of metabolites.^23^

Despite these promising insights, the mechanisms by which IF affects diabetic osteoporosis and the underlying processes remain unclear. This underscores the need to investigate how IF influences gut microbiota and its relationship to metabolic disease risk.^24–26^ Our study employed a comprehensive methodology, including gut microbiota analysis, exosome investigation, and genomic sequencing, to explore how exosomes from gut bacteria contribute to IF’s protective effects against diabetic osteoporosis. Confirmatory experiments included fecal transplantation, gut microbiota elimination assays, and exosome inhibition assays. Our findings demonstrate that IF modulating gut microbiota represents a promising therapeutic approach for metabolic degenerative skeletal disorders.

## Materials and methods

Both the institutional review board and our hospital’s ethics committee gave their approval for this research (DSYY-6420–22). Experiments involving animals must be carried out following the ARRIVE guidelines.

### Collection of gut microbiota

Fecal samples (100–150 g) were homogenized at a 1:5 ratio in sterilized normal saline (physiological saline supplemented with 0.05% l-cysteine-HCl). The resulting slurry was centrifuged at 6000 × g for 15 min at 4°C after passing through stainless steel sieves with pore sizes of 2.0, 1.0, 0.5, and 0.25 mm to remove undigested food and small particles. To get a final concentration of 10%, GM was reconstituted in half the initial volume of sterile, decreased saline and sterile glycerol after the supernatant was disposed of. It is anticipated that the full anaerobic procedure will take around an hour. To determine the concentration of bacteria, a small sample of each GM produced was subjected to agar plating. Escherichia coli (EC) and Christensenellaceae (Chr) samples from each donor were taken, divided into tiny aliquots, and either used immediately for further research or stored at −80°C for up to eight weeks before application to study the effect of bacteria on bone.

### Bacterial culture

As previously described, Christensenellaceae were grown in BHI broth (BD Bioscience, San Jose, CA, USA) with the addition of 0.5% porcine mucin (Sigma-Aldrich, St. Louis, MO, USA) and 0.05% l-cysteine-HCl (Sigma-Aldrich, St. Louis, MO, USA).^27^ E. coli ATCC25922 was grown in LB medium (MB2454, Meilunbio, Guangzhou, China) at 37 degrees Celsius. For E. coli and Chr. coli, bacteria were shaken at 170 rpm and then cultured either under anaerobic or aerobic conditions at 37°C. Bacterial colony-forming unit (CFU) counts were determined to estimate absorbance at 600 nm and calculate bacterial concentration.^28,29^

### Animals, DM brought on by STZ, and sporadic fasting

The STZ-induced DR and intermittent fasting studies were undertaken in adult male C57BL/6 Wild-type (WT) mice at eight weeks of age, as shown in Supplementary Figure S1. Mice were obtained from SLAM Laboratories. The mice were kept in both light and dark environments for 12 hours each. Each mouse was fed on AIN-93 M, a conventional diet purchased from TROPHIC Animal Feed Hi-Tech Co. The diabetic group received STZ injections (3 mg/kg/ICV) to induce hyperglycemia. The healthy control group was administered a vehicle under identical conditions. Blood glucose levels of 11.1 mmol/L or higher were considered hyperglycemic.

Four subgroups comprised the first animal batch: Vehicle, IF (intermittent fasting group), DM-Tx (diabetic osteoporosis group), and IF-DM-Tx (diabetes osteoporosis treated with intermittent fasting group). The STZ-induced DM mice were shown to have diabetic osteoporosis. However, for a total of 28 days, the IF mice were fed freely on the days in between after going without food for 24 hours every other day. Body weight, food, and water intake were measured on the fasting day. The animals were killed after radiologic testing and pathological staining so that tissues and serum could be extracted.

In the second set of animals, STZ-induced DM mice were separated into four subgroups (*n* = 9): control, DM-Tx(diabetic osteoporosis group), LG+DM-Tx(diabetic osteoporosis with LG supplement group), and Chr+DM-Tx(diabetic osteoporosis with Chr supplement group). The experiment used 1 × 10^8^ Escherichia coli (LG) and 1 × 10^8^ Christensenellaceae. Following the same IF protocol, the four groups were killed in order to collect intestinal, fecal, hippocampal, and serum samples for the multi-OMICS investigation. For RNA sequencing, metabolome analysis, and gut microbiome analysis, samples were taken from the hippocampus, serum, and gut microbiota, respectively.

As mentioned in the main text, stool samples from the second group of animals were selected for the third group and administered orally by gavage to 6-week-old Swiss Webster mice that were germ-free. Each fecal donor had one recipient mouse. Three cages per treatment were used, with mice housed four to a cage. In each experiment, the mouse was kept on a light/dark cycle of 12 hours and given autoclaved feed and drink at any time. Fecal pellets were taken once a week, and body weight and chow consumption were recorded. During the sacrifice, tissue from the tibia and spine was removed.

The four animals were placed into four groups: ABT-Control(antibiotic treatment group), ABT+DM-Tx(antibiotic treatment with diabetic osteoporosis group), ABT+Chr-DM-Tx(antibiotic treatment and then Chr supplement group), and ABT+LG-DM-Tx(antibiotic treatment and then LG supplement group). The program for the IF regimen was the same as the sets that were previously outlined. Beginning 14 days prior to the IF regimen and continuing during the experiment, the drinking water contained the antibiotic cocktail.^22^

The five sets of animal experiments: To test the role of EV secretion in Chr-induced bone health regulation, DM-Tx mice were fed 3 × 108 CFUs of GW4869-pretreated Chr orally. The four groups that made up the five sets of animals were Vehicle, DM(diabetic osteoporosis group), DM-Chr(diabetic osteoporosis with Chr supplement group), and DM-pre Chr(diabetic osteoporosis with GW4869-pretreated Chr supplement group).

For the six animal experiments, siRNAs targeting mouse miR-125 were prepared by Gima (Suzhou, China) for microRNA mimics and siRNA transfection, using scrambled siRNA as a negative control (NC). Cells were transfected with siRNAs at a concentration of 50 nm for 48 hours using Lipofectamine RNAiMAX Reagent (13778–100, Invitrogen, USA) after six hours of growth in Opti-MEM (31985–070, Invitrogen, USA). The Cy3-labeled miR-551b, NC, and miR-551b mimics were provided by RiboBio (Guangzhou, China). Cells were transfected using Lipofectamine 2000 reagent (11668030, Invitrogen, USA) for 24 hours at a terminal concentration of 50 nm.^30^ Four groups were formed from the animals in this set: Vehicle, DM (diabetic osteoporosis group), EVs (diabetic osteoporosis with extracellular vesicles group), and EVs-anti-miR (diabetic osteoporosis with extracellular vesicles treated with anti-miR551b group). When diabetic osteoporosis was present in the EVs-anti-miR group, 30 µL of adeno-associated virus (AAV) emulsion was injected into the right lateral femoral condyle. When diabetic osteoporosis was present in the EVs-anti-miR (DM + AAV-anti-miRNA-551b) group, 30 µL of an AAV-anti-miRNA-551b suspension was inoculated into the right lateral femoral condyle and the fourth lumbar vertebra.^31^

### Oral glucose tolerance test (GTT) and fasting blood glucose (FBG)

The insulin tolerance test, the glucose tolerance test, and the fasting blood glucose measurements were performed as described by Amir.^30^ After 12 weeks of treatment, mice received either an intraperitoneal (IP) injection of insulin (0.5 U/kg body weight) or a high glucose bolus (1 g/kg body weight). The mice were fasted for 16 hours on paper bedding. Then their blood glucose levels were measured. The glucose test was performed on mice that were either fasted (4–16 h) or fed (15 min to 1 h). As previously described, refeeding was achieved by IP injection of a soluble glucose bolus (1 g/kg body weight). The second drop of blood was collected by the use of a glucometer (Roche Diagnostics, Mannheim, Germany) after the first drop was discarded. Tail blood samples were taken at 0, 15, 30, 60, and 120 min after glucose administration.^32^

### µCT analysis

Dissected mouse femurs were preserved in 4% paraformaldehyde for one night prior to high definition μCT analysis (VIVACT 80; SCANCO Medical AG, Switzerland) according to the procedures of a previous study.^33,34^ The scanner was adjusted to a voltage of 70 kV and a current of 200 µA. The results showed that the isotropic voxel size was 55 kVp, the X-ray tube potential was 11.4 × 11.4 × 11.4 µm^3^, and the integration time was 400 ms. NRecon, CTAn v1.11, and μCTVol v2.2 were used to study the characteristics of the diaphyseal cortical bone and the trabecular bone of the distal metaphysics of the femur. The region of interest (ROI) for the trabecular bone study, excluding the growth plate and major spongiosa, began 0.3 mm proximal to the distal growth plate and continued proximally for 5% of the femoral length. The lower and upper limits for separating mineralized bone from other tissues were found to be 60 and 255, respectively. The ROI for cortical bone scanning was selected to start 40% of the length of the distal femur at the distal growth plate and extend 10% of the length of the femur proximally.^35^ ROIs were manually or semi-automatically traced by two blinded investigators to minimize bias, with inter-observer variability < 10%

### Analysis of histomorphometry, immunohistochemistry, and histology

Femora were prepared for histological and immunohistochemical labeling by decalcifying them for about three days at 4°C while being continuously shaken in 0.5 M EDTA (pH = 7.4). They were then fixed in 4% paraformaldehyde for 48 hours. The samples were dehydrated in ethanol and then embedded in paraffin. To identify OCN+ osteoblasts and TRAP+ osteoclasts across the entire ROI, which was also chosen for trabecular bone imaging, bone samples were longitudinally sectioned into 5 µm-thick slices and stained for OCN and tartrate-resistant acid phosphatase (TRAP) in accordance with the previously published protocol.^23^ The periosteal and endocortical surfaces of the cortical bone were not taken into account in the study. An Olympus C×31optical microscope (Olympus, Tokyo, Japan) was used to capture the pictures. The ROI’s secondary spongiosa cancellous bone’s whole surface was examined for osteoblast and osteoclast counts. Following that, the findings were normalized to the number of osteoblasts or osteoclasts per millimeter of the trabecular bone perimeter (N mm-1). By computing the osteoclast perimeter (OCs.PM) per millimeter of trabecular bone perimeter (Tb.PM) over the whole ROI, the size of osteoclasts on the secondary spongiosa cancellous bone surface was ascertained. The osteocalcin (OCN) primary antibody (Cat. No. gb11233; 1:200) and secondary antibody (Cat. No. gb23303; 1:200) were provided by Servicebio (Wuhan, China). We bought a Sigma-Aldrich TRAP staining kit (Cat. No. 387A-1KT).

To investigate dynamic bone formation, mice were given an intraperitoneal injection of 0.1% calcein (Sigma-Aldrich; 10 mg kg − 1 body weight) in PBS on days 9 and 3. Following collection, tibias were preserved for 48 hours in 4% paraformaldehyde, dried in progressively higher concentrations of ethanol, and then embedded in methyl methacrylate. A Leica DMI6000B fluorescence microscope (Solms, Germany) was used to generate and analyze slices of undecalcified bone that were 10 µm thick for calcein double labeling. Starting 0.3 mm distal to the proximal epiphyseal growth plate, the study zone extended 20% of the tibial length distally, excluding the growth plate and primary spongiosa but including the secondary spongiosa and cancellous bone. The periosteal and endocortical surfaces of the cortical bone were not taken into account in the study. Image-Pro Plus 6 software was used to assess the mineralized sedimentation rate (MAR) and bone resorption surface (BFR/BS) across the area of interest.^35^

### Tissue distribution of EVs in vivo

Following the manufacturer’s ex vivo fluorescent imaging instructions, several GM-EVs (total gut microbiota with extracellular vesicles group), LG-EVs (LG supplement with extracellular vesicles group), and Chr-EVs (Chr supplement with extracellular vesicles group) were labeled using the lipophilic dye DIR iodide (Santa Cruz). Using the previously mentioned methods, any possibly redundant fluorescent dye was eliminated from the DIR-labeled GM-EVs, LG-EVs, or Chr-EVs using bottom-up Optiprep density gradient centrifugation. The resulting EV-rich fractions were centrifuged for three hours at 100,000 × g and 4°C after being diluted with 30 mL of PBS. The EV pellets were reconstituted in PBS and administered intravenously (3 × 10^9^ EVs in 100 µL PBS), rectally (1.2 × 10^10^ EVs in 100 µL PBS), and orally (1.2 × 10^10^ EVs in 100 µL PBS) to all rats that had fasted for 48 hours. The mice in the vehicle received the same treatment as the mice in the control group. All of the mice were killed an hour later in order to remove their femora, tibias, muscles (especially the quadriceps femoris and triceps surae), livers, kidneys, and brains. Fluorescence tomography imaging equipment (FMT-4000; PerkinElmer, USA) was used to identify and quantify the fluorescent signals in these organs after they had been fixed for 15 minutes in 4% paraformaldehyde. The aforementioned three GM-EVs, LG-EVs, or Chr-EVs were also labeled with a green fluorescent dye (PKH67; Cat. No. MINI67; Sigma-Aldrich) in order to evaluate their tissue distribution. Mice were administered either 1.2 × 10^10^ vesicles containing GM-EVs, LG-EVs, or Chr-EVs, or 100 µL of PBS. The methods previously outlined were used to eliminate the excess color. The liver, kidney, brain, and femur tissues were extracted after an hour and preserved for four hours in 4% paraformaldehyde.

Femora were continually shaken in 0.5 m EDTA (pH = 7.4) for about three days at 4°C in order to decalcify them. The samples were all submerged in 30% sucrose for the whole night in order to dehydrate them. Before being sliced into slices that were 5 µm thick and immersed in an OCT compound (Sakura Finetek USA, Inc., Torrance, CA, USA), the samples were temporarily subjected to liquid nitrogen. The slices were stained with DAPI (0.5 µg mL^−1^; Invitrogen, Carlsbad, USA) after three five-minute PBS changes. To determine the average intensity of fluorescent signals, researchers used a confocal microscope (Leica SP8, Mannheim, Germany) and Image J 1.51j8 to take pictures. To assess the biodistribution of Chr-EVs using serum that included antibodies against Chr-EVs, they gave 1.2 × 10^10^ vesicles of Chr-EVs or an equal quantity of vehicle (100 µL PBS) to fasted mice for an hour. The femur, brain, kidney, and liver tissues of the mice were removed after they were killed and cut into the previously indicated 5 µm-thick slices. Serums from rabbits vaccinated with Chr-EVs were used to treat the sections overnight at 4°C after they had been blocked for 0.5 hours with 3% donkey serum. The slices were treated with rabbit preimmune serum as a negative control. The secondary antibody Alexa Fluor 488 AffiniPure Donkey Anti-Rabbit IgG (Cat. No. 711–545–152; Jackson ImmunoResearch; 1:300) was used to immunoreact with the sections after they had been cleaned. DAPI was used to stain the nuclei. Following the picture collection, the mean intensity for the positively stained regions was determined using Image J 1.51j8.^35^

### Serology

After being drawn into coagulation-promoting tubes, the blood is centrifuged for 15 minutes at 3000 rpm. After that, plasma is collected and stored at −80°C. The levels of ALP (Feimo, Beijing, China) and plasma lipopolysaccharide (LPS, J&W Pharmlab, Pennsylvania, USA) were determined using commercial enzyme-linked immunosorbent assay (ELISA) kits.

### Quantitative PCR

The distal left tibias were stored at −80°C after being flash frozen in liquid nitrogen. We crushed the frozen tibias under liquid nitrogen using a Bessman tissue pulverizer from Spectrum Laboratories in Rancho Dominguez, California, USA. Total RNA was isolated using Invitrogen’s TRIzol reagent (Carlsbad, CA, USA). We used the 2-ΔΔCT technique to analyze the relative change in gene expression.

### Western blotting

Western blotting was done after chondrocytes were cultivated at a density of 5 × 10^5^ cells per well in six-well plates and left to adhere for 48 hours. Each treatment group’s cells were isolated using RIPA lysis buffer (Boster, China, AR0102), which included 1% phosphatase inhibitor cocktail and 1% phenylmethylsulfonyl fluoride, after being on ice for 30 minutes. After that, the extract was collected and centrifuged at 12,000 g for 30 minutes at 4°C. Following centrifugation, each sample’s protein concentration was measured using a BCA assay kit (Boster, China, AR0146). After that, the supernatant was collected. Following SDS-PAGE separation, protein samples were transferred to PVDF membranes (Millipore, USA). After an hour at room temperature blocking with 5% skim milk, the membranes were subjected to a further hour at 4°C treatment with certain primary antibodies. After that, they received secondary antibody therapy for one hour at room temperature. The protein bands were photographed using a Bio-Rad scanner (Hercules, CA, USA) and visualized using a chemiluminescent reagent (Boster, China).^31^

### RNA‐sequence analysis

Invitrogen’s TRIzol reagent was used to sequence the total RNA that was isolated from chondrocyte samples. A Nanodrop spectrophotometer was used to measure the amount and quality of the extracted RNA samples. The integrity of the RNA was evaluated using the RNA Nano6000 Assay Kit on the Bioanalyzer 2100 system (Agilent Technologies, CA, USA). 1 μg of RNA was present in each sample and was used as the input for further analysis. Using the TaqMan MicroRNA Reverse Transcription Kit (4366596, Thermo Fisher Scientific) and Megaplex RT Primers (4399970, Thermo Fisher Scientific), cDNA was produced in subchondral bone after total RNA was extracted from chondrocyte samples. The 7900HT Real-Time PCR System was then used to conduct array analysis using the TaqMan MicroRNA Array Rodent Card A (4398967, Thermo Fisher Scientific) and TaqMan Universal PCR Master Mix (4352042, Thermo Fisher Scientific). The expression of U6 (△Ct values), which has a normal Gaussian distribution, was used to normalize the expression of microRNAs as indicated by Ct values. For group comparison, fold changes in the normalized data were then noted.^31^

### Evs uptake assay

According to manufacturer instructions, EVs were tagged with PKH26 (Catalog No. MINI26; Sigma-Aldrich). To get rid of excess dye, RAW264.7 cells were grown with labeled EVs (6 × 108 vesicles mL − 1) for three hours at 37°C. After treatment, the cells were fixed with 4% paraformaldehyde for 15 minutes and washed with PBS. Nuclei were stained with DAPI after being washed with PBS. The pictures were taken using a Carl Zeiss Axio Imager 2 fluorescence microscope (Germany).

### Extracellular vesicle purification

After chondrocytes were cultivated to about 100% confluence, extracellular vesicles were removed. After being centrifuged overnight at 100,000 g, they were grown for 48 hours in EV-depleted media, which is made from the supernatants of the whole medium. A differential centrifugation approach (300 × g for 10 min, 3000 × g for 10 min 10,000 × g for 20 min, and 100,000 × g for 70 min) was used to separate exosomes from supernatants at 4°C. The separated exosomes were centrifuged once more for 70 minutes at 100,000 × g after being washed with PBS. Dynamic light scattering (DLS) and a Nanosizer device (ZEN3790, Malvern Instruments, UK) were used in earlier studies to evaluate the size distribution of exosomes.^31,36^

The isolated exosomes on copper mesh were seen using transmission electron microscopy (TEM). The samples were cleaned with distilled water, dried, and dyed with phosphotungstic acid prior to TEM scanning.^31,37^

The Pierce BCA Protein Assay (23225, Thermo Fisher Scientific, USA) was used to measure the total protein content of exosomes. Lastly, PKH26 (MINI26; Sigma-Aldrich, USA) was used to tag the exosomes in accordance with the manufacturer’s instructions.

### Inhibition of exosomal release

The OME drug (PHR1059, Sigma Aldrich, USA) was made in DMSO and given to developing cells at a dose of 20 µg mL-1 for a whole day. The purpose of this activity was to prevent exosome (EV) release.^38^

### MicroRNA mimics and siRnas transfection

For use in siRNA and MicroRNA Mimic transfection, Gima (Suzhou, China) produced siRNAs against mouse miR-551b. Scramble siRNA was used as the negative control (NC) in this study. Before being transfected with siRNAs using Lipofectamine RNAiMAX Reagent (13778–100, Invitrogen, USA) for 48 hours at a concentration of 50 nm, the cells were cultured in Opti-MEM (31985–070, Invitrogen, USA) for six hours. The design work for the Cy3-labeled miR-551b, NC, and miR-551b mimics was finished by RiboBio (Guangzhou, China). Lipofectamine 2000 Reagent (11668030, Invitrogen, USA) was used to transfect the cells for 24 hours at a final concentration of 50 nm.^39^

### Adeno-associated virus (AAV)-anti-miRNA-551b preparation and analysis

Anti-miRNA-551b, an inhibitor of miRNA-551b, quickly lost its effectiveness in aqueous solutions. The chemical may have unidentified effects on various organs and systems, and pH and humidity affect systemic application. Accurate release is not guaranteed by local puncture or fractional administration. The most promising gene treatment technique at the moment is AAV. Over time, the enhanced safety profile endures.^40,41^ AAV9 is the AAV that is used, and its concentration is 1013 vg/ml. Based on our earlier studies, we created AAV-anti-miRNA-125-expressing green fluorescent protein (GFP) to aid in tracking. We previously synthesized AAV-anti-miRNA-125 and evaluated its functionality in cells. We discovered that a measurement of 30 µL is appropriate.^42^

### Transmission electron microscopy

Transmission electron microscopy was performed on carbon-coated copper grids with a 400 mesh size using EV preparations (10 µL). Next, the grids were washed three times with PBS (Electron Microscopy Sciences) right away. The grids were examined using transmission electron microscopy (TEM) (JEM 1010, JEOL, Japan) at 80 kV to produce TEM images after the extra solution was eliminated and allowed to dry.

### Histomorphometry

We evaluated secondary spongiosa to examine its dynamic features by intraperitoneally administering calcein (20 mg/kg) five and two days before sacrifice. Image-Pro Plus version 6.0 (Media Cybernetics Inc., US) and an Olympus B×61microscope (Olympus America, Inc., Center Valley, PA) were used to capture the images.

The left proximal tibia was histologically processed after 21 days of room temperature decalcification in 10% EDTA and a 24-hour fixation in 10% phosphate-buffered formalin. The proximal tibia was stained with hematoxylin and eosin (HE) and tartrate-resistant acid phosphatase. We used hematoxylin and eosin to stain the liver, spleen, and heart.

### 16 s rRNA gene sequencing

DNA was extracted from a 0.5 g fecal sample using the QIAamp Fast DNA Stool Mini Kit (Cat. No. 51604; Qiagen, Hilden, Germany). GM’s composition and relative abundance were assessed by transferring the DNA samples to GeneSky Biological Technology (Shanghai, China) for high-throughput 16S rRNA gene sequencing. The variable V4 region of 16S rDNA was amplified and sequenced using Illumina Miseq. Prerequisites for the quality filtering were a minimum length of 200 bp, a minimum quality score of 30, the lack of primer sequence mismatches, and a maximum of six ambiguous bases. Using USEARCH, duplicate sequences were screened, and chimeras were removed. The remaining sequences were compared to the Greengene database after being classified as operational taxonomic units (OTUs) using the UCLUST technique with a 97% similarity criterion. The relative abundance of the identified microbiota was assessed at the phylum, class, order, family, genus, and species levels. Bar graphs were used to display the relative abundance of bacteria at the genus and phylum levels.^31,35^

### Molecular modeling and docking

As previously stated, as part of our inquiry, we conducted a docking analysis of Adrβ1 and Abp1. The ChEMBL database was used to align the crystal structures of Adrβ1 and Abp1 (https://www.ebi.ac.uk/chembl/#). For the docking investigation, we used the CB-Dock program.^43^

### Statistical analysis

Statistical significance was established when the P-value was less than 0.05. The findings are shown using the mean ± standard deviation. The body weights from the time course investigation were evaluated using a two-way repeated-measure ANOVA. The Greenhouse-Geisser adjustment is applied to the data to reduce the likelihood that a significant result would be obtained just by coincidence. The 3 × 9 ANOVA was the result of the interaction between the intervention as a between-subject component and time (weeks 0–8 post-OVX+ABT stimulation) as a within-subject factor. Pairwise comparisons using Bonferroni correction were carried out for multiple comparisons of group means. The need for antibiotic medicine was suggested by a strong main impact of the intervention. The primary effects of time and intervention were analyzed in the data. GraphPad Prism 8.02 (La Jolla, CA, USA) was used to analyze the data using Tukey’s multiple tests and one-way ANOVA.

## Results

### Intervention with intermittent fasting impacts bone loss in diabetic mice

**Figure 1.**
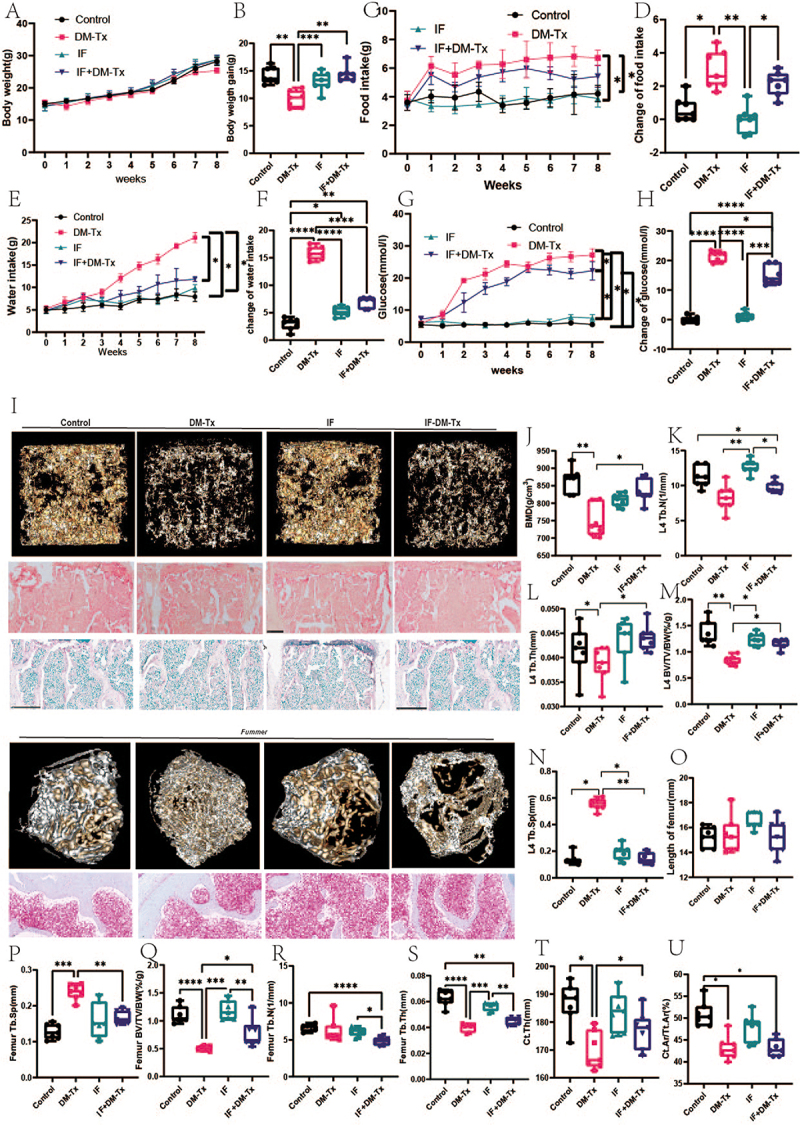
Intermittent fasting improves progress in diabetic osteoporotic mice. (A) body weight. (B) body weight gain. (C)food intake. (D)change of food intake. (E)water intake. (F)change of water intake. (G) serum glucose. (H) change of glucose. (I) Representative diagram of IF-treated diabetic osteoporotic mice. (J)BMD of fourth lumbar vertebrae. (K)tb.N of fourth lumbar vertebrae. (L)tb.Th fourth lumbar vertebrae. (M)BV/TV/BW of the fourth lumbar vertebrae. (N)tb.Sp of the fourth lumbar vertebrae. (O)length of femur. (P)tb.Sp of the femur. (Q) BV/TV/BW of femur. (R)tb.N of femur. (S)tb.Th femur. (T) Ct.Th femur. (U) Ct.Ar/Tt.Ar of femur. Data are presented as mean ± SEM, and statistical significance was determined by two-way ANOVA with Newman-Keuls multiple comparisons test, *n* = 12 mice per group, *p < 0.05, **p < 0.01.

As shown in Figure 1, we first examined the effects of intermittent fasting on the body weight, food intake, and blood glucose levels of diabetic mice. The body weight of the intermittent fasting intervention group increased dramatically in comparison to the group of diabetic mice. However, the diabetic mice showed no statistically significant improvement after treatment with intermittent fasting (Figure 1(A,B)). Despite the fact that intermittent fasting reduced food consumption, we found that diabetic mice ate much more than the control group. Compared to the control group, diabetic mice drank much more water. The diabetic group’s intermittent fasting schedules did not vary significantly.

**Figure 2.**
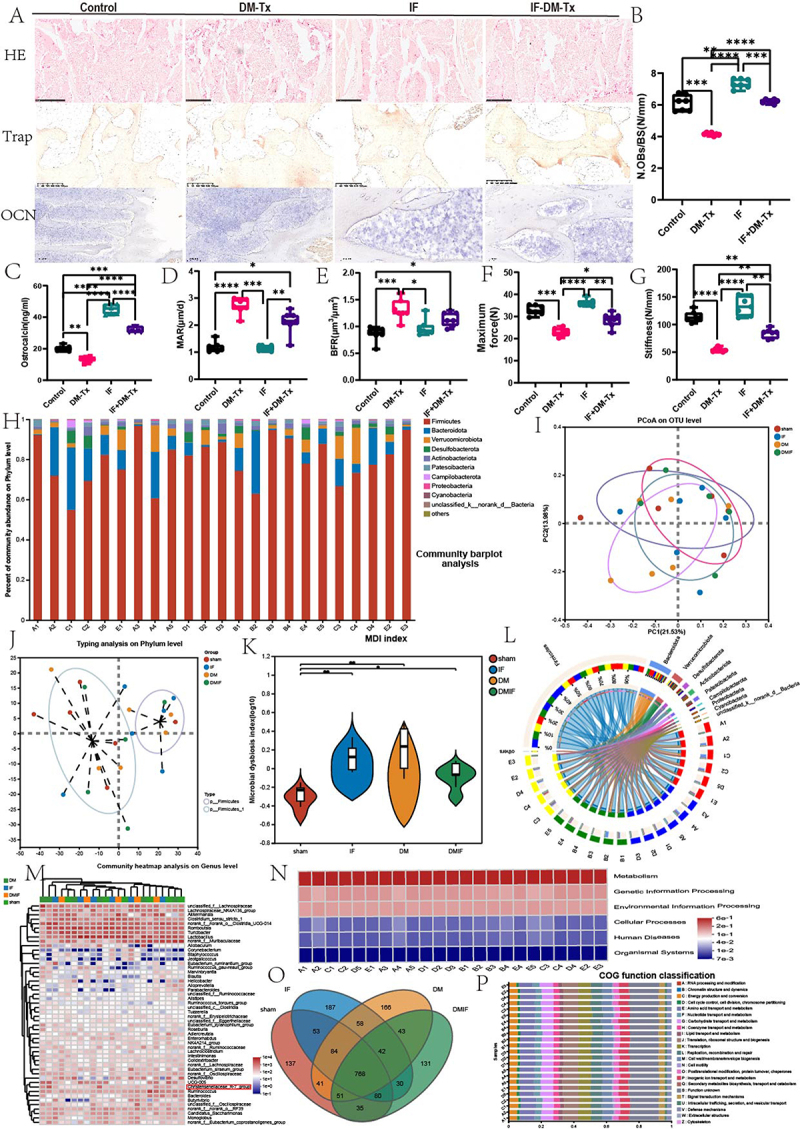
Intermittent fasting intervention modulates gut microbiota alterations in diabetic mice. (A) Representative diagram of HE stain, trap stain, and OCN immunohistochemistry stain.(B)N.Obs/BS.(C)ostrocalcin.(D)MAR.(E)BFR.(F)maximum force. (G) stiffness. (H)community barplot analysis. (I)principal cause analysis.(J)Typing analysis on phylum level. (K)microbiotal dysbiosis index. (L) correlation analysis of intestinal microbiota.(M) heatmap analysis.(N) feature enrichment analysis.(O) venn plot. (P) COG function classification. Data are presented as mean ± SEM and statistical significance was determined by two-way ANOVA with Newman-Keuls multiple comparisons test, *n* = 12 mice per group, *p < 0.05, **p < 0.01.

A mouse model of diabetes was effectively created two weeks after the intermittent fasting intervention, according to our analysis of blood glucose variations (Figure 1(C-F)). An intermittent fasting strategy dramatically lowered blood glucose levels in animals with diabetes (Figure 1(G-H)). Next, we examined the effects of intermittent fasting on the microstructure and lumbar bone mass in diabetic mice. After intermittent fasting, we found that BMD, Tb.Th, and BV/TV dramatically increased in diabetic mice, but Tb.N and Tb.Sp did not change substantially (Figure 1(I-O)). Significant changes were observed in the femoral side Tb.sp. (*p* = 0.01), BV/TV (*p* < 0.001), and Ct.Th (*p* < 0.05), as shown in Figure 1(P-U). The outcomes of the mechanical analysis, osteogenic effects, and osteoclast staining were next examined. The findings demonstrated that whereas BFR and MAR levels sharply decreased, OBs/Bs, maximum force, and stiffness were all markedly elevated by the intermittent fasting intervention (Figure 2(A-G)). These findings imply that intermittent fasting improves glucose control and bone mass in diabetic mice.

### Intermittent fasting improves the abundance of Chr bacteria in diabetic osteoporotic mice

We also looked at the gut microbiome’s changes after the intermittent fasting trial since we believe it might have a significant effect. The microbial composition of these specimens was determined by high-throughput sequencing of 16S rRNA gene (16S rDNA) amplicons. At the phylum, class, order, family, genus, and species levels, the composition of the microbiota was examined. When compared to the diabetes group, the intermittent fasting intervention resulted in significant decreases in Bacteroides microbacteria and Leucobacterium recursum and large increases in Christensenellaceae and Tenericutes at the phylum level. Figure 2(H) shows the histogram, Figure 2(I) shows the PCA plot, Figure 2J shows the phylum level, Figure 2(K) shows the microbiota dysbiosis index (MDI) value, and Figure 2(L) shows the categorization map, while Figure 2(M) shows the heat map. According to quantitative real-time PCR (qRT-PCR) research that focused on the variable region of the 16S rRNA gene of Chr.768 species, which are present in all four groups based on the Ven plot data (Figure 2(O)), the Chr bacterial abundance was significantly increased by intermittent fasting intervention in comparison to diabetic mice. The results of the functional enrichment analysis showed that metabolic and RNA regulatory pathways were essential to the process (Figure 2(N,P)). The results of our investigation showed that during the intermittent fasting intervention, the quantity of the Christensenellaceae microbiota grew dramatically. Taking supplements of Christensenellaceae bacteria also greatly delays the development of diabetic osteoporosis.

**Figure 3.**
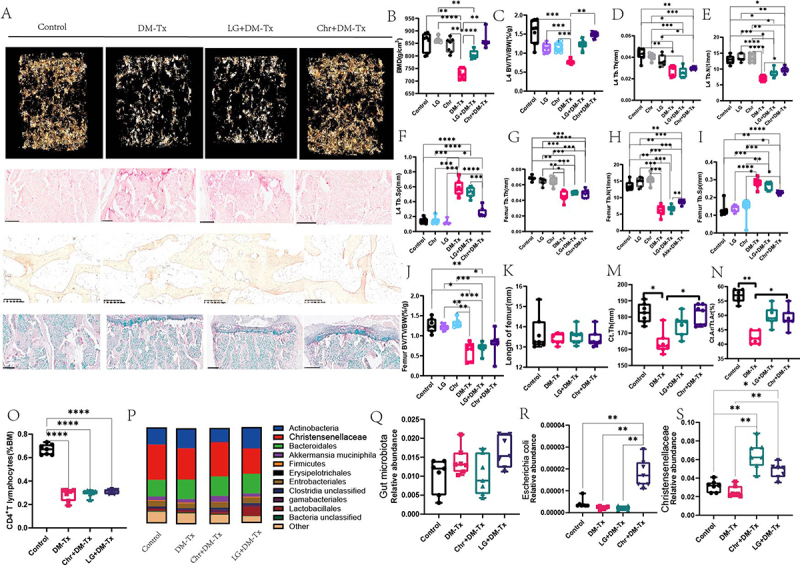
Effects of Chr supplementation on diabetes-induced osteoporosis. (A) Representative diagram of bone microstructure, HE stain, OCN stain. (B)BMD of the fourth lumbar vertebrae. (C)BV/TV/BW of the fourth lumbar vertebrae. (D)tb.Th of fourth lumbar vertebrae. (E)tb.N of fourth lumbar vertebrae. (F)tb.Sp of the fourth lumbar vertebrae. (G) Tb.Th of femur. (H)tb.N of femur. (I)tb.Sp of femur. (J) BV/TV/BW of femur. (K)length of femur. (T) Ct.Th femur. (U) Ct.Ar/Tt.Ar of femur. (O)CD4+ T lymphocytes. (P) histogram of intestinal microbiota composition. (Q)the total load of gut microbiota. (R) Escherichia coli relative abundance. (S)christensenellaceae relative abundance. Data are presented as mean ± SEM, and statistical significance was determined by two-way ANOVA with Newman-Keuls multiple comparisons test, *n* = 12 mice per group, *p < 0.05, **p < 0.01.

After comparing the effects of Chr and LG on bone, we looked into whether giving diabetic osteoporosis mice direct supplements of Chr bacteria every two weeks for eight weeks had an impact on changes in bone mass in comparison to giving them LG supplements, which contain bacteria that are frequently found in the stomachs of warm-blooded animals and people.^43^ Oral administration of live Chr bacteria significantly lowers bone loss in diabetic osteoporosis, according to DXA and Micro-CT investigations. Additionally, it reduces lumbar and femoral Tb.SP while increasing lumbar and femoral Tb.N and BV/TV, as well as femoral Tb.N, Ct.Th, and Ct.Ar/Tt.Ar, According to bone microscopic data (Figure 3(A-N)). However, the LG supplement did not seem to have any discernible impact on bone mass or microstructure in diabetic osteoporosis. Chr was repeatedly shown to be superior to LG in the bone length analysis and three-point bending test. Therefore, it was possible to make the bones of DM mice longer and stronger.

Next, the impact of supplementing with Chr on osteogenesis and osteoclastogenesis was examined. The results showed that diabetic mice supplemented with Chr had a greater osteogenic response than diabetic osteoporotic mice. These results were examined by immunohistochemistry labeling and an ELISA test employing the bone formation marker OCN. While osteoblast data decreased, LG supplementation did not affect OCN levels. Chr, but not LG, was provided, according to the TRAP staining data. In diabetic mice given Chr, bacteria reduced bone resorption activity, osteoblast survival, and osteoclast development. New bone growth and mineralization increased in diabetic mice given calcified green fluorescent double standard. On the other hand, Chr supplementation led to a noteworthy trend of decreased BFR/BS and MAR values in comparison to diabetic mice (Supplement Figure 2). Chr supplementation has a major impact on the immune system, according to earlier research. Likewise, we observed a significant reduction in CD4+ T cells in the Chr-supplemented group (Figure 3(O)). Lastly, we looked at the gut microbiota changes shown in the bar graph and found that there was no improvement in the overall gut microbiota percentage (Figure 3(P)). Figure 3(Q-S) displays the LG and Chr bacteria. We discovered that the LG supplement greatly raised the bacterial abundance in both groups. According to the research mentioned above, taking a supplement of Chr may help treat osteoporosis in people with diabetes by increasing osteogenesis and reducing osteoclasts.

### Experiments with fecal transplantation and elimination experiments give more evidence that intermittent fasting improves the development of diabetic osteoporosis

**Figure 4.**
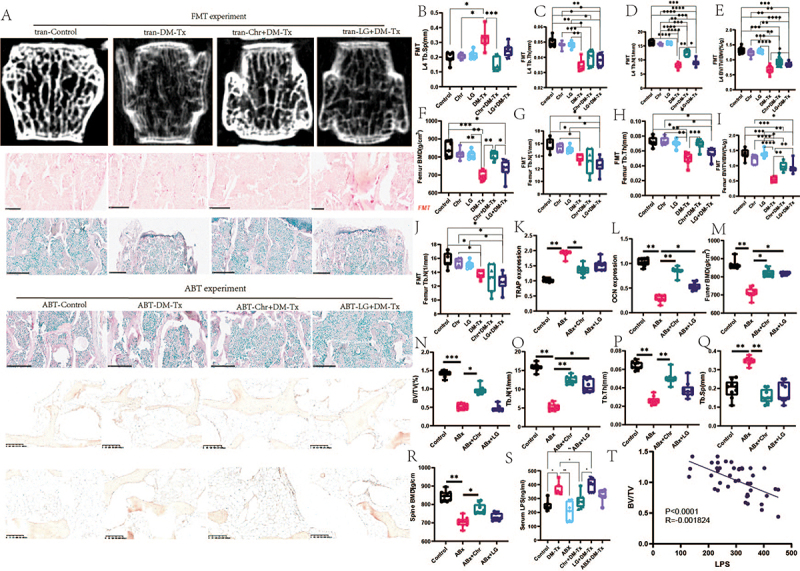
Effects of fecal transplantation assays and gut microbiota elimination assays on diabetes-mediated osteoporosis. (A) Representative diagram of bone microstructure, HE stain, OCN stain of fecal transplantation assays, and gut microbiota elimination experiment. (B)tb.Sp of the fourth lumbar vertebrae. (C)tb.Th of fourth lumbar vertebrae. (D)tb.N of fourth lumbar vertebrae. (E)BV/TV/BW of the fourth lumbar vertebrae. (F)BMD of the fourth lumbar vertebrae. (G)tb.Sp of femur.(H) Tb.Th femur. (K)TRAP expression. (L)OCN expression. (M) femur BMD in gut microbiota elimination assays. (N) BV/TV/BW of femur in gut microbiota elimination assays. (O)tb.N of femur in gut microbiota elimination assays. (P) Tb.undefined.The femur of in gut microbiota elimination assays. (Q) Tb.Sp of femur in gut microbiota elimination assays. (R) BMD of the fourth lumbar vertebrae in gut microbiota elimination assays. (S)serum LPS. (T) correlated BV/TV with LPS level. Data are presented as mean ± SEM, and statistical significance was determined by two-way ANOVA with Newman-Keuls multiple comparisons test, *n* = 12 mice per group, *p < 0.05, **p < 0.01.

Gut microbiota is often confirmed by fecal transplantation testing and gut microbiota elimination assays.^22,23^ In mice with control, diabetic osteoporosis, LG supplementation, and Chr supplementation, we first examined fecal transplantation. Mice that have had their excrement supplemented with LG are transformed into germ-free mice. The Chr-supplemented feces transplant with diabetic osteoporosis group showed a large increase in BV/TV and Tb.N and a significant decrease in Tb.Sp, as shown in Figures 4(A-E). The following outcomes were obtained from the fecal transplantation experiment. According to femur bone microstructure analyses, the Chr supplementation group had significantly higher BMD, Tb.Th, and BV/TV (Figure 4(F-J)).

Using antibiotics are used for mice, we conducted further gut microbiota eradication experiments. According to the results, osteoblast markers like OCN significantly increased and osteoclast indicators like TRAP significantly decreased after the removal of the gut microbiota and subsequent Chr bacteria supplementation (Figure 4(K-L)). Furthermore, in terms of tibia BMD and bone microstructure, the Chr supplementation group markedly raised BMD, BV.TV, Tb.N, Tb.Th, and lowered Tb.Sp levels after gut microbiota removal (Figure 4(F-J)). Similar findings were observed for the bone density of the lumbar spine. BV/TV in Figure 4(N) is increased in the Abx-LG-treated group and remains unchanged, indicating that LG has no effect on bone mass in Abx-treated mice. After that, we tracked LPS levels and found that the Chr bacteria treatment drastically reduced LPS levels when compared to diabetic mice. After that, we found a significant inverse association between BV/TV and LPS (*R* = −0.001824, *p* < 0.0001). The effectiveness of Chr supplementation in preventing diabetic osteoporosis was shown by both the Chr supplementation experiment and the gut microbiota elimination test.

### EV secretion is necessary for Chr’s anti-diabetic osteoporotic effect

We preincubated Chr colonies with GW4869, a neutral sphingomyelinase (nSMase) inhibitor that limits EV release and stops Chr colonies from secreting EVs, in order to investigate the function of EVs in Chr-induced anti-osteoporosis. We next investigated the potential that the mice would be protected against OVX-induced osteoporosis by these GW4869-pretreated CHr, administered by gastric gavage twice a week for eight weeks. Prevent diabetes-induced osteoporosis in mice. The effect of GW4869 on the viability of Chr colonies was determined by counting the number of bacterial colonies on YCFA agar plates. The number of bacterial colonies on YCFA agar plates four days after GW4869 inoculation did not differ significantly from vector-treated Chr flora, indicating that GW4869 did not influence the viability of the bacteria. Normalizing EV levels to the total concentrations of all EV proteins is a common and simple method of measuring the concentration of EVs, which are determined by their proteins. The total protein content of retrieved EVs sharply dropped after four days of GW4869 treatment, suggesting a significant suppression of EV synthesis in Chr. The EVs protein levels in GMs pretreated with GW4869 were considerably lower than in the vector-pretreated group, even after GW4869 was taken out of the medium and the treatment Chr was cultivated for four additional days. We counted the number of particles in the Chr EVs treated with either vector or GW4869 using the nanoparticle tracking method (NTA). The amount of EV particles in Chr was significantly reduced following a 4-day treatment with GW4869; this decrease continued even after GW4869 was removed from the culture for 4 days. This outcome is consistent with changes in EVs’ protein composition. These results suggest that GW4869 suppresses EV secretion for a long period of time.

**Figure 5.**
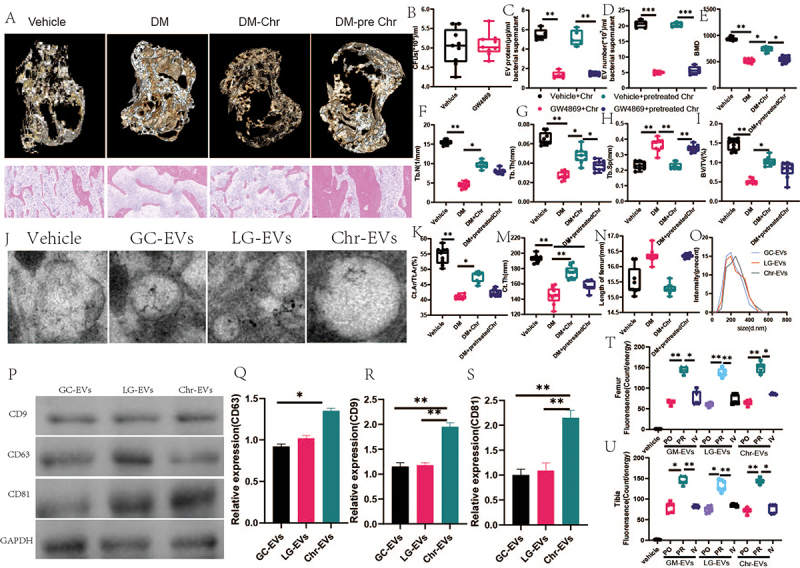
Chr-derived exosomes may play a role in regulating diabetic osteoporosis. (A) Representative diagram of bone microstructure and HE stain. (B) the total load of gut microbiota. (C) total protein contents and D) particle numbers of EVs from GM treated with vehicle or GW4869 for 4 days, or from GM pretreated with vehicle or GW4869 for 4 days and cultured in fresh medium without vehicle or GW4869 for another 4 days. *n* = 3 per group. (E)BMD of the fourth lumbar vertebrae. (F)tb.N of fourth lumbar vertebrae. (G)tb.Th of fourth lumbar vertebrae. (H)tb.Sp of the fourth lumbar vertebrae. (I)BV/TV/BW of the fourth lumbar vertebrae. (J) Representative diagram of exosome electron microscopy results. (K) Ct.Ar/Tt.Ar of femur. (M) Ct.Th of femur. (N)length of femur.(O) morphological analysis. (P-S)EVs markers of CD9, CD63, and CD81. (T-U) femur and tibia fluorescence. Data are presented as mean ± SEM, and statistical significance was determined by two-way ANOVA with Newman-Keuls multiple comparisons test, *n* = 12 mice per group, *p < 0.05, **p < 0.01.

According to micro-CT imaging, Chr may raise bone microstructure and BMD levels in diabetic mice while also increasing bone mass and microarchitecture. TV/BV, Tb. N, TB. Th, Th. Ar/Tt. Ar, Ct. Th and Ct. Here are such instances. However, both Tb.Sp. are suppressed. Furthermore, while both Chr-treated and vector-treated diabetic mice with GW4869 pretreatment showed similar amounts of bone mass and microstructural characteristics, Figure 5 demonstrates that the bone-protective actions of Chr depend on the release of EVs.

To determine whether pasteurization (70°C, 30 min) is a common method for reducing the number of microorganisms in liquids and dairy products, we used bacterial colony counting assays on plates to assess the viability of Chr and nano particle tracking assays to count the number of particles in Chr-derived EVs from pasteurized or viable Chr. Pasteurized Chr did not prevent mice from losing bone as a result of diabetes, according to prior research by Lawenius et al..^44^ T As shown in Figure 5, the complete reduction in Chr-derived EV production and the 100% mortality of Chr following pasteurization suggest that the decreased production of Chr-EVs may be the cause of pasteurization’s inability to produce the skeletoprotective benefits noted in the Lawenius et al. study.^45^

**Figure 6.**
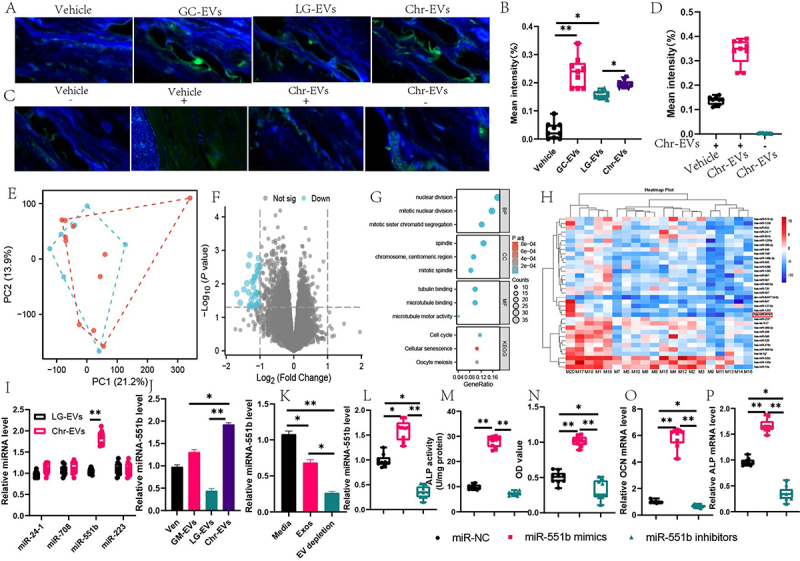
Chr source mir-551b in the regulation of osteoclast activity. (A) confocal microscopy analysis of the femoral sections from mice treated with the PKH67-labeled EVs for 1 h by oral route(top line) and Representative images of the *Chr*-EVs antibody (ab)-stained(bottom line). (B-C) quantification of the fluorescent signals in PKH67-labeled EVs and *Chr*-EVs antibody (ab)-stained. (E) Principal cause analysis of miRNA analysis. (F) volcano map miRNA analysis. (G) KEGG. (H) heatmap of miRNA analysis. (I) relative of four miRNA levels. (J) relative of miR-551b level in EV. (K)relative of miRNA-551b level after depletion of EV. (L) relative of miR-551b level after treatment with miR-551b mimics and inhibitors.(M)ALP activity. (N) OD value. (O) relative OCN mRNA. (P)relative ALP mRNA. Data are presented as mean ± SEM, and statistical significance was determined by two-way ANOVA with Newman-Keuls multiple comparisons test, *n* = 12 mice per group, *p < 0.05, **p < 0.01.

Following their separation from the Chr medium, we characterized the EVs using dynamic light scattering (DLS) and transmission electron microscopy. GM, LG, and Chr EVs were spherical or cup-shaped, with corresponding dimensions of 215.02 ± 48.66 nm, 199.57 ± 52.61 nm, and 166.88 ± 35.99 nm. These values were similar to previously reported observations of bacterial EVs. Following the assignment of the E designation, the ability of GM-derived EVs, LG-derived EVs (LG-EVs), and Chr-EVs containing DIR iodide to permeate host tissues was evaluated. In vitro fluorescence imaging revealed that mice with DIR-labeled GM-derived EVs (GM-EVs) had high fluorescence signals in their tibia and femur. For one hour, COL-derived extracellular vesicles (LG-EVs) were administered orally, intravenously, or rectally (Figure 5(T-U)). The exosomes were then stained with the markers CD9, CD63, and CD81. As shown in Figure 5(P), our results demonstrated a significant increase in the number of exosomes produced by Chr. Those were CD9, CD63, and CD81. It has been shown that mouse bone tissues, including the periosteum, endosteum, cortical bone, trabecular bone, and bone marrow, contain PKH67-labeled GM-EVs, LG-EVs, and Chr-EVs. This implies that by specifically targeting osteoblasts, these EVs may have an impact on bone metabolism. Figure 6(A-B) illustrates its existence. The average fluorescence intensity demonstrated the ability of the three GM-EV, LG-EV, and Chr-EV types to translocate to mouse bone tissue.

We also utilized sera that had Chr-EVs-antibodies and collected sera from rabbits that had received injections of Chr-EVs to see whether oral Chr-EVs might be present in mouse tissues. Figure 6(B,C). shows that mice treated with vector and Chr-EVs displayed fluorescent signals in the kidney, liver, brain, and bone. It’s interesting to note that mice treated with vectors showed much stronger signals in these organs than animals treated with vector-treated Chr-EVs. This indicates that Chr-EVs may be transported to host tissues under physiological settings and that oral administration of exogenous Chr-EVs increases their concentration in those tissues.

### Exosomes made from chromosomes emit miR-551b, which is essential for avoiding osteoporosis in diabetics

To ascertain which miRNAs are essential for exosome function, we next used exosome sequencing. PCA plots are shown in Figure 6(E), and volcano plots and heatmap findings are shown in Figure 6(F-H). These results demonstrate that Chr-secreted exosomes have a substantial expression of miR-551b. When we validated the high-expression miRNA expression in all four sequencings, we only discovered a substantial increase in miR-551b expression. We next looked at the expression of miR-551b in colony exosomes from diabetic mice, LG supplement colony exosomes, and Chr supplement colony exosomes. We discovered that the expression of miR-551b was much higher in the Chr supplement colony exosomes. We observed a significant decrease in miR-551b content after removing the EVs (Figure 6(E-H)).

**Figure 7.**
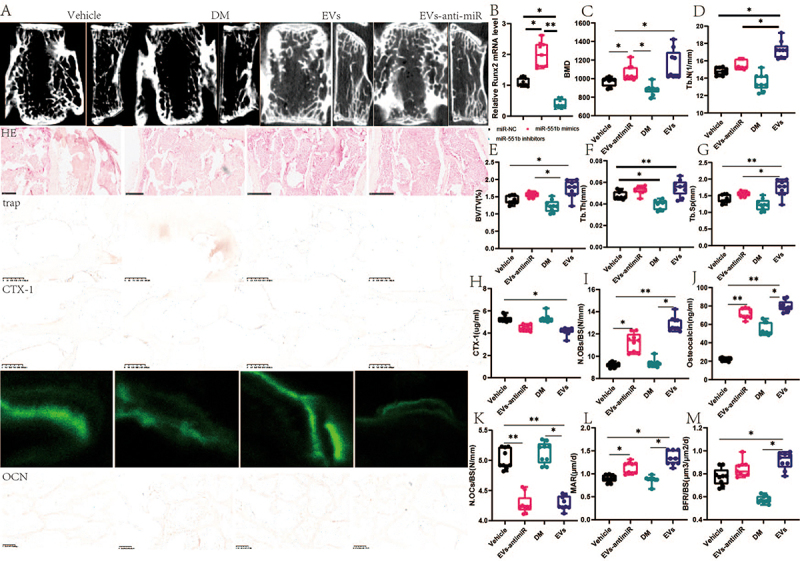
Reversal of EVs amelioration diabetic osteoporosis after miR-551b inhibition. (A) Representative diagram of bone microstructure and HE stain.(B) relative Runx2 mRNA. (C)BMD of the fourth lumbar vertebrae. (D)tb.N of fourth lumbar vertebrae. (E)BV/TV/BW of the fourth lumbar vertebrae. (F)tb.Th fourth lumbar vertebrae. (G)tb.Sp of the fourth lumbar vertebrae. (H)CTX-1. (I) N.Obs/BS. (J)osteocalcin. (K) N.Obs/BS. (L)MAR. (M) BFR/BS. Data are presented as mean ± SEM, and statistical significance was determined by two-way ANOVA with Newman-Keuls multiple comparisons test, *n* = 12 mice per group, *p < 0.05, **p < 0.01.

In the group that was activated, miR-551b levels would soar, but in the group that was inhibited, they would fall. We next assessed alterations in ALPase activity and found that activation of miR-551 markedly enhanced cellular activity and ALPase activity; mRNA levels demonstrated significant increases in OCN, RUNX2, and ALP levels (Figures 6(I-P) and 7(B)).

Furthermore, we simulated the molecular fitness of ALP and MiR-551b using the docking model, and the results are shown in Supplementary Figure S2, which suggests that both ALP and miR-551b have high spatial fitness. We then conducted in vivo research to confirm the function of miR-551b generated from exosomes. The results demonstrated that EV intervention led to large reductions in Tb.Sp, CTX-1, and N.OCs/BS levels and significant increases in BMD, Tb.N, BV/TV, OCN, N.Obs/BS, MAR, and BFR. The suppression of miR-551b reversed this process. Additionally, according to this data section, EV-derived miR-551b may be a key target for intermittent fasting treatments intended to cure diabetic osteoporosis.

## Discussion

The immune and endocrine systems are among the many bodily systems that are significantly impacted by intermittent fasting.^6–9,11^ Intermittent fasting has been shown in many trials to avoid alterations in gut flora.^17,18,46^ Three primary lines of new results emerged from our work. First, we found that in diabetic mice, osteoporosis production was decreased by intermittent fasting. Second, we discovered that the Chr bacteria in the gut microbiota promote bone density and strength and may play a key role in controlling the anti-osteoporotic effects of diabetes. Third, by transferring functional EVs and their source, miR-551b, from bacteria to osteoblasts across extended distances, we propose a novel mechanism for communication between gut microbiota and bone. Our findings imply that during intermittent fasting therapies for diabetic osteoporosis, gut microbiota enriched with Chr bacteria may be significant. This route produces Chr-derived EVs and their source, miR-551b, which aid in bone homeostasis maintenance in diabetic mice.

Diabetes-induced bone loss is one of the main causes of osteoporosis. Two aspects of diabetes that may be detrimental to bone are hyperglycemia and the accumulation of advanced glycosylation end products. T1D has relatively low endogenous insulin levels, while T2D has hyperinsulinemia and insulin resistance.^47^ These changes in insulin status distinguish T1D from T2D. A meta-analysis of 66 studies found that compared to controls, individuals with T1DM and T2DM had substantially higher levels of sclerostin and significantly lower levels of osteocalcin and C-terminal cross-linking telopeptides. Although there was no appreciable difference between T1DM and control participants, TRAP levels were significantly lower in T2DM patients than in controls.^48^ Low blood levels of osteocalcin and type I collagen carboxy-terminal peptide β-specialized sequences have been found in people with type 1 and type 2 diabetes, suggesting low bone conversion rates.^49,50^ T2DM patients had considerably smaller cortical area and width, despite the fact that there was no change in trabecular features between the groups.^51^ Dynamic indices revealed considerably reduced T2DM values for the mineralized surface, bone production rate, bone-like surface, and osteoblastic surface.^52^ This may be explained by reduced bone resorption due to the inhibition of osteoclast development by oral hypoglycemic drugs (e.g., enteral insulinotropic medicines) and slower bone matrix maturation and mineralization rates caused by the hyperglycemic diabetic environment.

The miR-551b is implicated in regulating osteoblast differentiation and bone formation through multiple pathways. In cancer studies, miR-551b directly suppresses cyclin D1 (a key cell cycle regulator) by binding to its mRNA 3‘UTR, thereby inhibiting cell proliferation.^53,54^ This mechanism may extend to osteoblasts, where miR-551b could modulate osteogenic precursor cell proliferation by downregulating cyclin D1, thereby fine-tuning bone matrix synthesis. The miR-551b shows a negative correlation with CDK6 and SETD2 in cancer models.^55,56^ While direct evidence in osteoblasts is limited, miR-551b’s role in other tissues suggests it may target genes like RUNX2 or Osterix (key osteogenic transcription factors), which warrants further investigation.

The microbial interactions involving Christensenellaceae and other gut microbiota species have been investigated in previous studies.^27,57^ Bacteroides-derived propionate and Christensenellaceae-associated butyrate could synergistically suppress pro-inflammatory cytokines (e.g., TNF-α) that drive osteoclastogenesis.^58,59^ In addition, addressing niche competition for mucin degradation or carbohydrate utilization may indirectly influence bone remodeling by altering intestinal barrier integrity or systemic inflammation.^60,61^ SCFA production by Christensenellaceae, Bacteroides, and Lactobacillus might activate GPR43/41 receptors on osteoblasts, promoting Wnt/β-catenin signaling.^62^ The potential cross-talk between Bacteroides-mediated bile acid modification and Christensenellaceae-associated FXR/TGR5 signaling, which regulates osteoclast differentiation.^63^ In addition, to contextualize Christensenellaceae within broader microbial communities, we will analyze our 16S rRNA sequencing data to construct a Correlation analysis of intestinal microbiota (Figure 2(L-M)). This will identify key taxa (e.g., Bacteroides) that correlate with Christensenellaceae, providing ecological insights into their functional partnerships or antagonisms. Finally, we are also concerned current focus on single taxa and propose future studies using gnotobiotic models colonized with defined consortia (e.g., Christensenellaceae + Lactobacillus) to dissect their combinatorial effects on bone density.

Our data suggest that the observed miR-551b induction is primarily mediated by IF-driven remodeling of gut microbiota and associated metabolic shifts, rather than the elimination of specific nutrients. The differential expression of microRNAs and lncRNAs, including miR-15b, miR-103–2, miR-302a, miR-6985, and miR-5624, was regulated by IF, which exerts significant anti-inflammatory and immunoregulatory effects.^64^ Zhou et al found that MiR-27a-3p may participate in CIH-induced IR by modulating the PPAR γ/IRS1/PI3K/AKT signaling pathway. The time-restricted eating regimen downregulated miRNA, which, in turn, could inhibit the pathways of cell growth and activate the pathways of cell survival and might promote healthy aging.^65^ We agree that distinguishing between the effects of intermittent fasting (IF) itself versus nutrient exclusion is critical to interpreting our findings.

Intermittent fasting is effective in treating a variety of illnesses, including diabetes, cancer, heart disease, and neurological disorders, including stroke, Parkinson’s disease, and Alzheimer’s disease.^66,67^ Our previous study indicates that anti-osteoporotic drugs like alendronate or antidiabetic drugs like metformin can only treat osteoporosis or diabetes on their own, and that the combined effects of osteoporosis and diabetes are not particularly favorable for diabetic osteoporosis.^32^ Intermittent fasting has been shown to successfully reduce body weight and improve many health indicators, including insulin resistance and the reduction of risk factors for cardiovascular disease.^68,69^ DNA repair, autophagy, and the activation of adaptive cellular stress signaling pathways are some of the cellular and molecular mechanisms that promote health and prevent illness.^9,70^ Our results imply that the development of diabetic osteoporosis is slowed by an intervention that involves intermittent fasting.

Several lines of evidence link intermittent fasting (IF), microbiota-dependent free fatty acid (FFA) metabolism, and systemic metabolomic reprogramming. IF significantly enriched Christensenellaceae and Lachnospiraceae – bacterial families with well-documented roles in short-chain fatty acid (SCFA) production.^71^ IF can improve metabolic health through a variety of mechanisms, including enhancing insulin sensitivity, lowering blood pressure, and improving blood lipid levels.^72–74^ IF can significantly influence fatty acid metabolism by regulating processes such as fatty acid breakdown, oxidation, and synthesis, thereby promoting metabolic reprogramming.^75–78^ In addition to its effects on metabolic health, intermittent fasting (IF) has received widespread attention for its modulation of the neuroimmune microenvironment.^79^ The neuroimmune microenvironment refers to the complex network of interactions between the nerves and the immune system, which plays a key role in neuroinflammation, neuroprotection, and repair.^80^ IF modulates neuroimmune responses by influencing metabolites derived from fatty acid metabolism, such as ketone bodies and short-chain fatty acids (SCFAs).^81,82^ For example, ketone bodies reduce oxidative stress and protect neurons by activating the Nrf2 pathway, while SCFAs regulate neuroinflammation by regulating T cell and microglia activity.^83,84^ IF significantly influences metabolic state and neuroprotective mechanisms by activating signaling pathways such as AMPK and SIRT1, which promote fatty acid breakdown while inhibiting synthesis.^85^ IF also plays a key role in neuroprotection by regulating mitochondrial function, reducing oxidative stress, and balancing autophagy, apoptosis, and ferroptosis.^86,87^ For example, IF has shown potential in the prevention and treatment of neurodegenerative diseases by reducing the production of reactive oxygen species (ROS) and enhancing the antioxidant system to alleviate oxidative damage to neurons.^88,89^ What’s more, IF can counteract disease processes and improve functional outcomes, including diabetes, cardiovascular disease, cancer, and neurological conditions.^90,91^ The cellular and molecular mechanisms by which IF improves health and counteracts disease processes include activation of adaptive cellular stress response signaling pathways, DNA repair, and autophagy that enhance mitochondrial health.^92,93^

In our studies, transferring IF-adapted microbiota (enriched in Christensenellaceae) to AL-fed mice significantly increased serum EV miR-551b levels, even in the absence of fasting. This demonstrates that gut microbiota remodeling is sufficient to induce miR-551b packaging into EVs, independent of host dietary cycles. While microbial EVs deliver miR-551b, the potential host cell is also involved in this process. As a signaling small molecule that regulates the body’s metabolism, miRNAs can be released through a variety of cells in a variety of ways. For example, a mouse model of middle cerebral artery occlusion (MCAO) injected with miR-100-5p-loaded hNPC-derived exosomes exhibits a smaller size of cerebral infarction, reduced apoptosis, and improved neurological function.^94^ Sugantha et al found that RNA cargo of sEVs changes during malignancy, with specific miRNAs driving Ductal carcinoma in situ progression. Re-expression of miR-205 offers a promising therapeutic approach to prevent DCIS from becoming invasive.^95^ L. lactis-miR-146b-induced reduction in intestinal inflammation was partially dependent on EVs that contained miR-146b, which modulated the activation of classically activated macrophages (M1 macrophages).^96^ Faecalibacterium prausnitzii EVs upregulate the expression of key proteins involved in HR repair, such as BRCA1 and BRCA2, thereby reducing DNA damage and inhibiting the cGAS-STING pathway, which is central to the inflammatory response.^97,98^

The phylum Thick-walled Bacteria includes the newly identified family Christensenellaceae, which is becoming more important for human health. The strongest correlation between the human gut and metabolic illnesses to yet has been found to be between the relative abundance of Christensenellaceae in the gut and host body mass index (BMI), which is adversely associated across numerous populations and research.^99^ The relationship between metabolic disease and the ecology of human gut microorganisms is the most robust and long-lasting one discovered so far.^57^ Numerous other illnesses have also been connected to the family, such as inflammatory bowel disease and obesity.^100^ Compared to healthy controls, the flavonoid Lactobacillus cumberum was shown to be more prevalent in depressive illnesses; nevertheless, the centered Log-Ratio abundance of Christensenellaceae associated with health was much lower. These results were associated with elevated oxidative stress and systemic low-grade inflammation.^101^ Older Italians with a richer Christensenellaceae gut microbiota have been associated with a better metabolic state and less visceral fat tissue.^102^ According to Hou et al., this study demonstrates a causal link between osteoporosis and the Christensenellaceae family, with blood levels of 3,4-dihydroxybutyrate serving as a minor mediator.^103^ LG and Chr may not fully reverse DM-induced decreased BV/TV in femur under the tested experimental conditions, micro-CT analyses (Figure 3(G-I)) and serum biomarkers (OCN in supplementary figure S2) revealed that lumbar BMD, BV/TV, and Tb. N was significantly increased in the Chr-DM group and decreased significantly in the lumbar and femoral Tb.Sp than the DM group. In addition. Our preliminary time-course analyses also indicate that LG and Chr may exert transient protective effects during earlier stages of bone remodeling, which could be attenuated by the progressive nature of DM-related metabolic dysregulation. In many clinical studies, the gut microbiome has been shown to have a profound impact on bone quantity, quality, and overall strength.^24–26^ The gastrointestinal tract contains the highest concentration of immune cells that communicate with the microbial community and trigger the release of metabolites or immune responses that directly affect the immune system.^27,28^ In bone immunology studies, the balance of Th17/Treg and related inflammatory cytokines has been shown to be closely related to bone metabolic disorders.^30^ While the data indeed show comparable total T cell numbers across the DM-Tx, LG+DM-Tx, and Chr+DM-Tx groups, we propose the following interpretations to this observation that Chr supplementation may not be significantly associated with T cell-related immune responses. By positively altering gut microbiota bacteria, our results, which demonstrate the importance of Christensenellaceae in diabetic osteoporosis, also imply that supplementing with this plant may be a highly attractive treatment option.

Interventions such as intermittent fasting may impact diabetic osteoporosis by modifying the gut microbiome. The synthesis of fatty acids, bile acids, and the promotion of vitamin synthesis are some of the mechanisms of action of gut microbiota on diabetic osteoporosis that are now being studied.^104^ Additionally, it has been linked to the production of osteoclastogenic components and the suppression of CD4 T-cells.^105^ Bacterial EVs carry payloads from their parent bacterium, such as proteins, peptidoglycans, lipopolysaccharides, and nucleic acids. The EVs carry these chemicals into the host cell, altering the receiving cell’s biological activity.^106^ Furthermore, compounds associated with cell membranes that are beneficial to host health, such as vitamin K2, certain fatty acids, and miRNAs, may infect bacterial EVs.^29^ A previous study that demonstrated sympathetic intervention improves the intervention of exosome-derived miR125 in the development of osteoarthritis supports the critical role of miRNAs as essential mediators for EV delivery.^31^ MiR-144-3p may induce ferroptosis by preventing osteosarcoma cells from proliferating, migrating, and invading by suppressing ZEB1 expression.^107^ Using miR-874-3p to modulate necroptosis, HucMSC-Exos may promote healing and reduce damage to renal tubular epithelial cells. In order to reduce kidney damage, this might result in novel therapeutic strategies and ideas for treating AKI and the AKI to CKD transition pathway.^108^ MiR-146a-3p generated from serum exosomes improved macrophage M2 polarization in allergic rhinitis by targeting VAV3 via the PI3K/AKT/mTOR pathway. According to these findings, VAV3 and miR-146a-3p may be good targets for developing novel treatments for allergic rhinitis.^109^ We provide a novel mechanism by which bacteria transfer functional EV-derived miRNAs to osteoblasts, hence promoting communication between the microbiota and the host skeleton. We discovered that EV secretion is required for GM-induced anti-osteoporotic effects, and that oral administration of either GM-EVs or Chr-EVs markedly improved bone mass and strength in mice with diabetes-related osteoporosis. Later studies confirmed that these EV-derived miR-551b regulate ALP activity to improve bone metabolism. These findings suggest that GM could interact with host osteoblasts by releasing EVs that cross the host’s intestinal barrier, enter the somatic circulation, and eventually enter bone tissue. Furthermore, it is proposed that EV-derived miR-551b directly affects osteoclast bone production and resorption. These results are supported by data demonstrating that these EVs directly stimulate osteogenesis while inhibiting osteoclastogenesis in vitro. Chr-EVs’ beneficial effects on bone health are further enhanced by the possibility that this component has anti-osteoclastogenic or bone-enhancing qualities. More study is needed to identify the factors determining the bone-protective properties of these EVs.

## Limitations

There were some limitations in our study. First of all, this study has a small sample size, mainly focuses on basic experiments, and lacks the verification of clinical research results. Second, our study focused on one bacterial change in the microbiota and confirmed the interactions between the microbiota and between the microbiota and the diathesis through correlated validation. In addition, the study employs diabetic mice, which may not fully recapitulate human gut-bone axis dynamics due to species-specific differences in microbiota composition, immune responses, or bone turnover rates, and STZ-induced diabetic osteoporosis focuses on hyperglycemia-driven bone loss, potentially overlooking contributions from other osteoporosis subtypes. In addition, our study deliberately prioritized establishing the functional hierarchy of the gut-bone axis in diabetic osteoporosis, where we confirmed that miR-551b supplementation rescues bone microarchitecture while its inhibition exacerbates osteoclastogenesis. However, the deeper molecular characterization of how Christensenellaceae-derived extracellular vesicles (EVs) and miR-551b modulate bone cell activity needs to be further studied. Finally, Exosomes derived from gut microbiota can repair organ and tissue damage. In addition, it also has the advantages of long half-life, low immunogenicity, good stability, and the ability to cross the blood-brain barrier, and has shown excellent performance and potential practical applications in bone diseases.^110^ However, exosomes derived from intestinal microbiota still have weaknesses such as insufficient targeting ability and poor therapeutic effect, and may require a large number of doses to ensure efficacy.^111,112^ Therefore, how to engineer exosomes derived from intestinal microbiota is an important method for future research.^113,114^

## Conclusion

Diabetes-induced osteoporosis is mediated by EVs-miR-551b carrying Christensenellaceae via intermittent fasting. This might provide fresh insights into the mechanisms behind dietary treatments that slow the course of diabetic osteoporosis.

## Provenance and peer review

Not commissioned, externally peer-reviewed

## Abbreviation

DM: diabetes mellitus
IF: intermittent fasting
GM: gut microbiota
Chr: Christensenellaceae
LG: Escherichia coli
CFUs: colony forming units
AAV: adeno-associated virus
FBG: Fasting blood glucose
GTT: oral glucose tolerance test
IP: intraperitoneal
GM-Evs: total gut microbiota with extracellular vesicles group
LG-Evs: LG supplement with extracellular vesicles group
Chr-Evs: Chr supplement with extracellular vesicles group
BFR/BS: bone resorption surface
MAR: Mineralized sedimentation rate
OVX: ovariectomy mice

## Supplementary Material

Supplemental Material
