# Supplementary material for: Intermittent fasting triggers interorgan communication to improve the progression of diabetic osteoporosis
Source: Gut Microbes. 2025 Sep 30;17(1):2555619. doi: 10.1080/19490976.2025.2555619 (PMC12490001; doi:10.1080/19490976.2025.2555619)
Supplement: Supplemental Material [file KGMI_A_2555619_SM5426.docx]

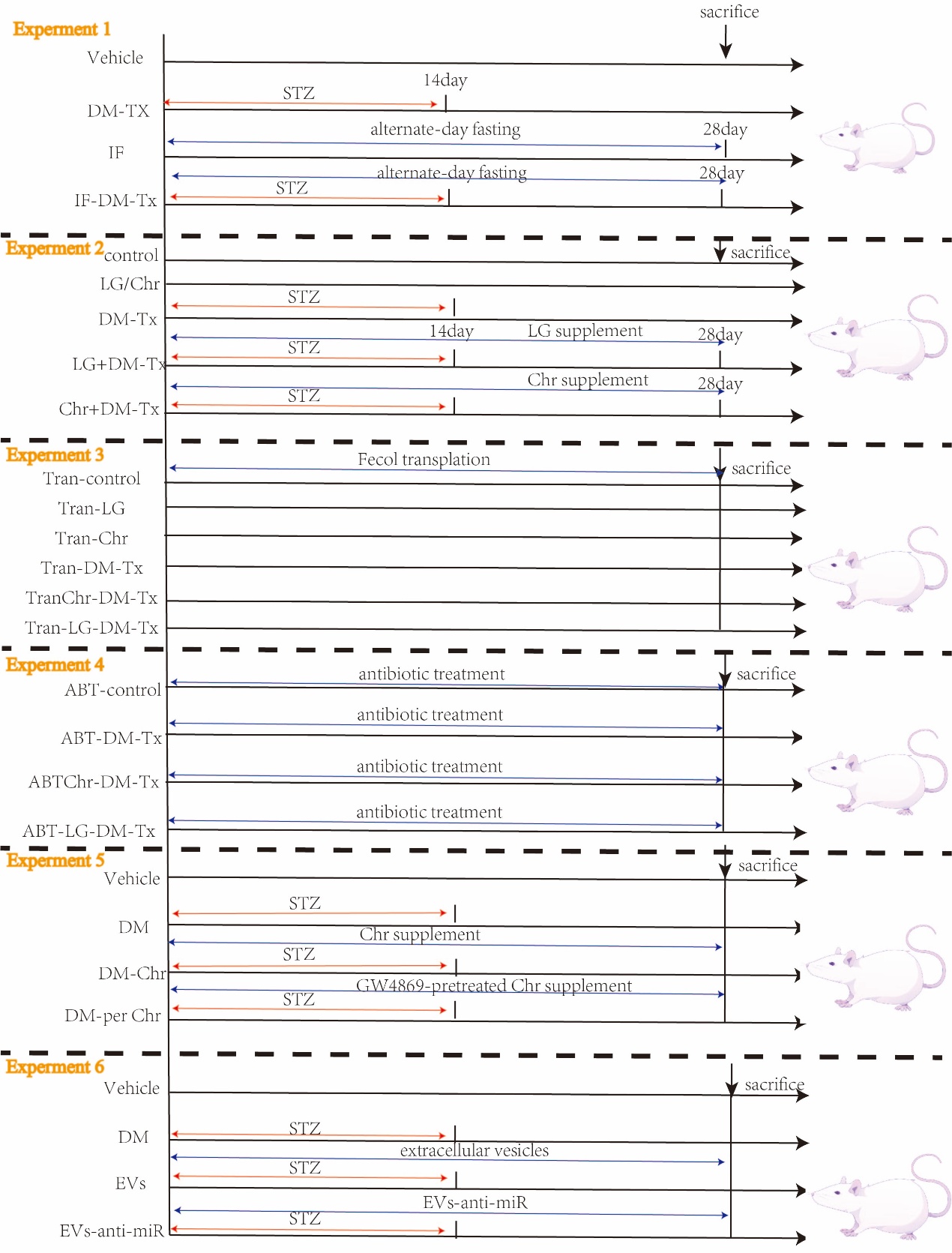


Supplement figure1. Experimental flow chart


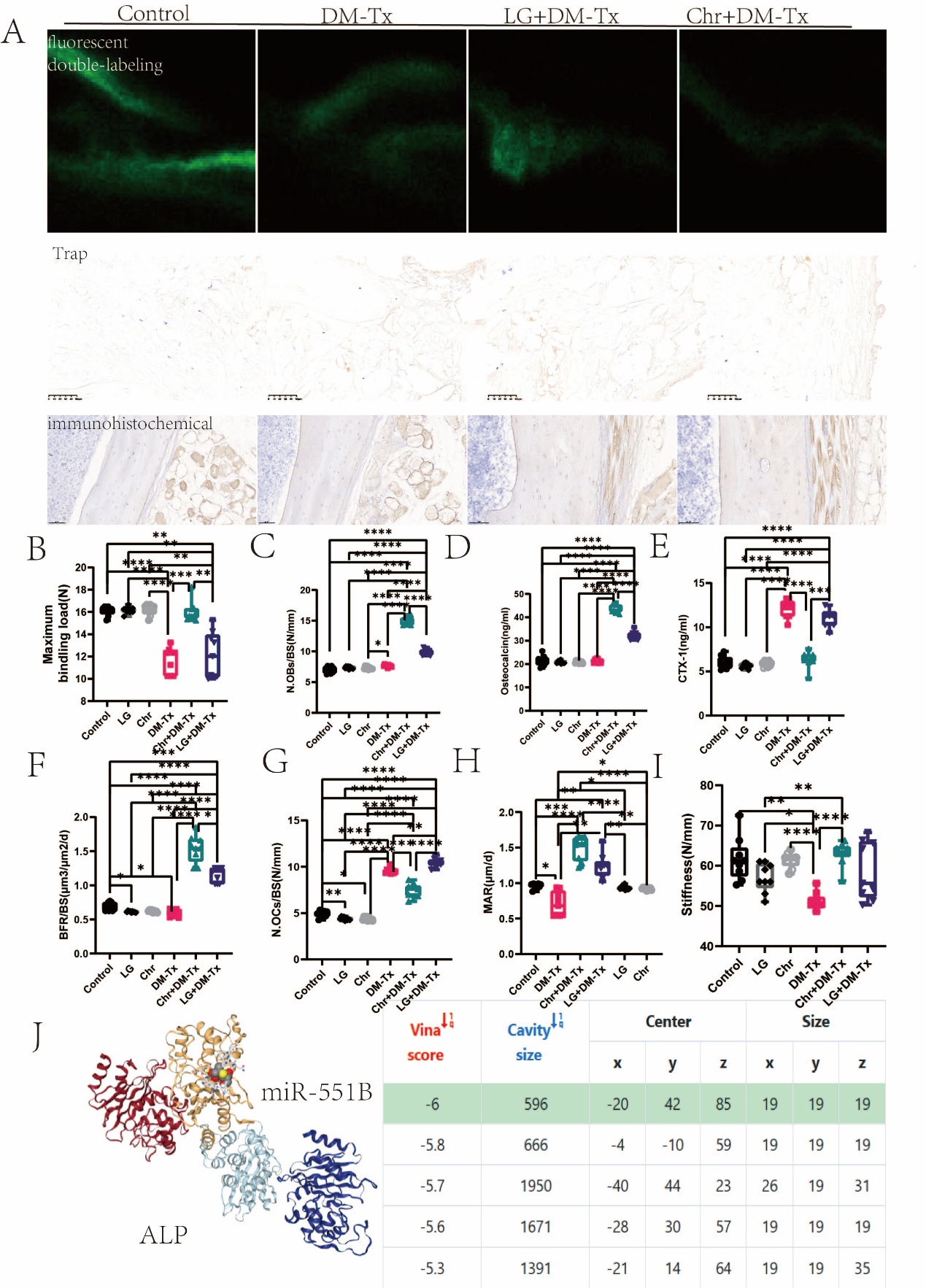


Supplement figure2. Effects of Chr supplementation on osteoblasts and osteoclasts and the Docking model. (A) Representative Diagram of Fluorescent double standard, Immunohistochemistry, Trap staining.(B)maximum binding load. (C)N.Obs/BS. (D)Osteocalcin. (E)CTX-1. (F) BFR/BS. (G) N.Obs/BS. (H)MAR. (I) Stiffness. (J) docking model between miR-551b and ALP. Data are presented as mean ± SEM, and statistical significance was determined by two-way ANOVA with Newman-Keuls multiple comparisons test, n = 12 mice per group, *p < 0.05, **p < 0.01.


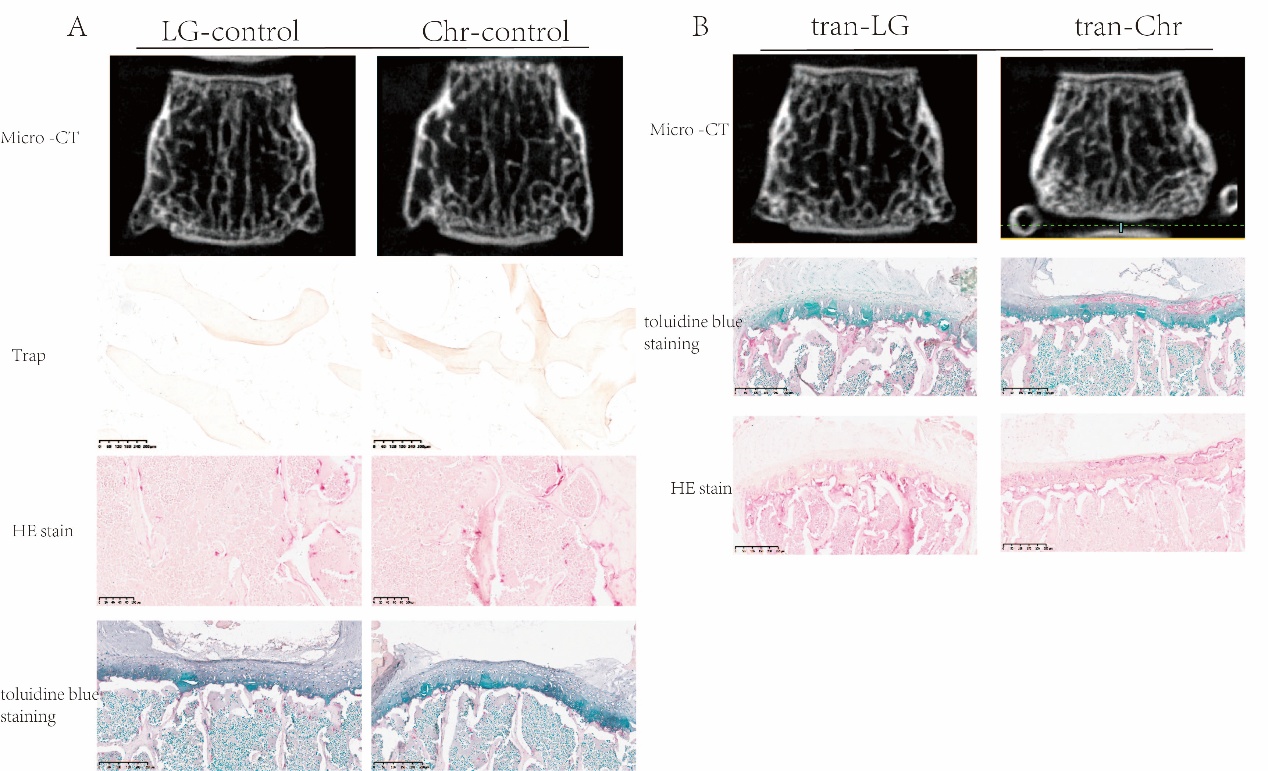


Supplement figure3. Representative Diagram of LG and Chr supplement experiment and fecal transplant experiment with LG and Chr.
